# Risk prediction model for long-term atelectasis in children with pneumonia

**DOI:** 10.1186/s12890-023-02464-x

**Published:** 2023-05-15

**Authors:** Yonghan Luo, Yanchun Wang, Kenan Gong

**Affiliations:** 1 Second Department of Infectious Disease, Kunming Children’s Hospital, Kunming, 650000 Yunnan China; 2 Department of Respiratory Diseases, Children’s Hospital of Chongqing Medical University, Chongqing, China

**Keywords:** Bronchoscopy, Prediction model, Atelectasis, Children

## Abstract

**Background:**

This study aimed to develop a risk prediction model for long-term atelectasis in children with pneumonia.

**Methods:**

A retrospective study of 532 children with atelectasis was performed at the Children’s Hospital of Chongqing Medical University from February 2017 to March 2020. The predictive variables were screened by LASSO regression analysis and the nomogram was drawn by R software. The area under the Receiver Operating Characteristic (ROC) curve, calibration chart and decision curve were used to evaluate the predictive accuracy and clinical utility. 1000 Bootstrap resampling was used for internal verification.

**Results:**

Multivariate logistic regression analysis showed that clinical course before bronchoscopy, length of stay, bronchial mucus plug formation, age were independent risk factors for long-term atelectasis in children. The area under the ROC curve of nomogram was 0.857(95% CI = 0.8136 ~ 0.9006) in training set and 0.849(95% CI = 0.7848–0.9132) in the testing set. The calibration curve demonstrated that the nomogram was well-fitted, and decision curve analysis (DCA) showed that the nomogram had good clinical utility.

**Conclusions:**

The model based on the risk factors of long-term atelectasis in children with pneumonia has good predictive accuracy and consistency, which can provide a certain reference value for clinical prevention and treatment of long-term atelectasis in children.

## Introduction

Atelectasis is defined as the reduction of volume or air content of one or more lung tissues due to a variety of reasons, resulting in functional tissue collapse or atrophy, alveolar hypoxia and pulmonary vasoconstriction, improper ventilation-perfusion matching; all of these ultimately affect lung function [1]. The most common cause of atelectasis is infection [2, 3]. However, the pathogens and lesion sites are different in children with atelectasis. Bronchoscopy is an important treatment option for atelectasis in children [4], [3]. It can help directly observe the pathological changes of the local bronchial surface, and promote the recovery of atelectasis through bronchoalveolar lavage (BAL) and etiological examination of bronchoalveolar lavage fluid (BALF), greatly improving the diagnosis and treatment of atelectasis in children. Studies [5–7] have shown that bronchoscope interventional therapy can improve the overall effective rate of treatment, shorten the length of hospital stay, and promote the imaging resolution of pulmonary lesions in children with atelectasis. However, some atelectasis cannot be cleared with medical treatment alone and a single BAL, resulting in long-term atelectasis. And long-term atelectasis easily leads to recurrent infection, bronchiectasis, bronchiolitis obliterans, and lung necrosis [8]. Considering the above factors, this study retrospectively analyzed 532 children with atelectasis to identify the pulmonary location of lesions, bronchoscopic changes, and etiological differences in children with atelectasis, and attempted to explore the risk factors of long-term atelectasis and develop a clinical prediction model to provide a basis for clinicians to prevent and treat long-term atelectasis.

### Data source

In this study, clinical data of 532 children with pneumonia associated with atelectasis were collected from the Respiratory Center of the Children’s Hospital of Chongqing Medical University from February 2017 to March 2020. The study protocol was approved by the Ethics Review Committee of the Children’s Hospital of Chongqing Medical University. Due to the retrospective design of the study, the requirement for informed consent was waived, and all patient information were processed anonymously.

### Study population

The inclusion criteria were as follows: (1) hospitalized children under 18 years of age;(2) hospitalized with respiratory symptoms, such as cough, fever, and/or shortness of breath;(3) diagnosed with pneumonia [9]; (4) had a CT examination that showed atelectasis. The exclusion criteria were as follows: (1) history of congenital heart disease, hematologic malignancy, immunodeficiency, or immunosuppressant use; (2) serious lack of clinical data;(3) atelectasis caused by other factors such as bronchial foreign body; tuberculosis, or bronchial compression;(4) loss of follow-up.

All children with atelectasis received their first bronchoscopy and BAL after they were diagnosed. According to the results of imaging examination one month after their discharge, those cases with reopening atelectasis were classified as reopening group, and the cases with chest CT showing no re-opened atelectasis were classified as the non- reopening group.

### Study variables and data extraction

The basic data of the 532 children were collected from the electronic medical records at the Children’s Hospital of Chongqing Medical University, which included demographic characteristics (including sex, age, weight, and length of hospital stay), pulmonary localization of atelectasis, and etiological results of BALF. *Respiratory syncytial virus, adenovirus, influenza virus, and parainfluenza virus* were detected by direct fluorescence immunoassay. In addition, sputum culture and polymerase chain reaction (PCR) were used to detect bacteria and *M. pneumoniae*. Finally, abnormal manifestations by bronchoscopy (bronchomalacia, tracheal or bronchial stenosis, tracheal bronchus, bronchiectasis, suppurative change, nodular changes, bronchial mucus plug (BMP) formation, airway hyperresponsiveness), clinical course before BAL were collected.

### Statistical analysis

Excel software was used to record the data and R software (4.2.2 Version) was used to analyze the data. variables with p < 0.05 in the univariate analysis were selected into LASSO logistic regression model to explore the risk factors of long-term atelectasis in children with pneumonia. all cases were divided into train (70% data) set and validating set (30% data) by using “Sample ()” function in R software. The selected predictors are used to develop a predictive model represented as a nomogram. 1000 Bootstrap resampling was used for internal verification. The area under the receiver operating characteristic (ROC) curve, calibration chart and decision curve analysis were used to evaluate the accuracy, consistency and clinical utility of the prediction model. Statistical significance was set at P < 0.05.

## Results

### Clinical features of included patients

A total of 532 children with infective atelectasis were included, including 264 boys (49.6%) and 258 girls (51.4%). The mean age was 52.83 ± 40.02 months in the reopening group and 66.34 ± 37.98 months in the non-reopening group. The average body weight of the reopening group was 17.21 ± 9.49 kg and that of the non-reopening group was 19.61 ± 8. 40 kg.The mean length of hospital stay was 6.61 ± 3.77 days in the reopening group and 9.46 ± 7.48 days in the non-reopening group. Atelectasis was most commonly located at the right middle lobe and right upper lobe (35.5% and 23.3%, respectively). There were 55 cases of tracheal or bronchial stenosis (10.3%), 32 cases of nodular changes (6.0%), 27 cases of BMP formation (5.1%), 23 cases of airway hyperresponsiveness (4.3%), 22 cases of suppurative change (4.1%), 13 cases of bronchiectasis (2.4%), 10 cases of bronchomalacia (1.9%), and 2 cases of granulation hyperplasia (0.4%). Among the pathogens detected by BALF, the four pathogens with the highest detection rates were *Mycoplasma pneumoniae* (225 cases, 42.3%), *Adenovirus* (69 cases, 13%), *Streptococcus pneumoniae* (64 cases, 12%), and *Haemophilus influenzae* (44 cases, 8.3%). A total of 134 cases (25.2%) could detect multiple (≥ 2) pathogens. Table 1 shows the clinical features of the two groups.

**Table 1:** The clinical characteristics between reopening group and non-reopening groups

	Overall (n = 532)	Reopening group (n = 383)	Non- reopening group (n = 149)	*P*
**Clinical feature**				
sex, n (%)	264 (49.6)	199 (52.0)	65 (43.6)	0.103
Age, (mean (SD)), m	56.61 (39.89)	52.83 (40.02)	66.34 (37.98)	< 0.001
Weight, (mean (SD)), kg	17.88 (9.26)	17.21 (9.49)	19.61 (8.40)	0.007
Length of stay, (mean (SD)), d	7.41 (5.24)	6.61 (3.77)	9.46 (7.48)	< 0.001
Clinical course before bronchoscopy, (median [IQR])	11.00 (8.00, 17.00)	10.00 (7.00, 13.00)	17.00(13.00, 30.00)	< 0.001
**Pulmonary localization of atelectasis**				
Left upper lobe, n (%)	101 (19.0)	72 (18.8)	29 (19.5)	0.958
Left lower lobe, n (%)	91 (17.1)	53 (13.8)	38 (25.5)	0.002
Right upper lobe, n (%)	124 (23.3)	99 (25.8)	25 (16.8)	0.035
Right middle lobe, n (%)	189 (35.5)	135 (35.2)	54 (36.2)	0.909
Right lower lobe, n (%)	75 (14.1)	43 (11.2)	32 (21.5)	0.004
multiple lobes, n (%)	90 (16.9)	55 (14.4)	35 (23.5)	0.017
**Bronchoscopic changes**				
Bronchial mucus plug formation,n (%)	27 (5.1)	10 (2.6)	17 (11.4)	< 0.001
Airway hyperresponsiveness, n (%)	23 (4.3)	12 (3.1)	11 (7.4)	0.054
Nodular changes, n (%)	32 (6.0)	25 (6.5)	7 (4.7)	0.553
Bronchomalacia, n (%)	10 (1.9)	6 (1.6)	4 (2.7)	0.619
Tracheal or bronchial stenosis, n (%)	55 (10.3)	46 (12.0)	9 (6.0)	0.061
Granulation hyperplasia, n (%)	2 (0.4)	2 (0.5)	0 (0.0)	0.924
Bronchiectasis, n (%)	13 (2.4)	8 (2.1)	5 (3.4)	0.591
Suppurative change, n (%)	22 (4.1)	10 (2.6)	12 (8.1)	0.01
**Pathogens of BALF**				
Multiple pathogens, n (%)	134 (25.2)	95 (24.8)	39 (26.2)	0.829
*Mycoplasma pneumoniae*, n (%)	225 (42.3)	148 (38.6)	77 (51.7)	0.008
*Adenovirus*, n (%)	69 (13.0)	53 (13.8)	16 (10.7)	0.417
*Parainfluenza virus*, n (%)	15 (2.8)	15 (3.9)	0 (0.0)	0.031
*Influenza virus*, n (%)	19 (3.6)	13 (3.4)	6 (4.0)	0.926
*Respiratory syncytial virus*, n (%)	17 (3.2)	10 (2.6)	7 (4.7)	0.34
*Haemophilus influenzae*, n (%)	44 (8.3)	34 (8.9)	10 (6.7)	0.523
*Streptococcus pneumoniae*, n (%)	64 (12.0)	50 (13.1)	14 (9.4)	0.309
*Klebsiella pneumoniae*, n (%)	5 (0.9)	5 (1.3)	0 (0.0)	0.368
*Escherichia coli*, n (%)	3 (0.6)	3 (0.8)	0 (0.0)	0.661
*Moraxellacatarrhalis*, n (%)	7 (1.3)	5 (1.3)	2 (1.3)	1
*Stenotrophomonasmaltophiliastrain*, n (%)	1 (0.2)	0 (0.0)	1 (0.7)	0.624
*Serrati amarcescens, n (%)*	1 (0.2)	1 (0.3)	0 (0.0)	1
*Acinetobacter baumannii, n (%)*	7 (1.3)	7 (1.8)	0 (0.0)	0.216
*Pseudomonasaeruginosa, n (%)*	5 (0.9)	4 (1.0)	1 (0.7)	1
*Staphylococcus aureus, n (%)*	14 (2.6)	9 (2.3)	5 (3.4)	0.727

### The predictors for non-reopening in the training set

**Fig. 1:**
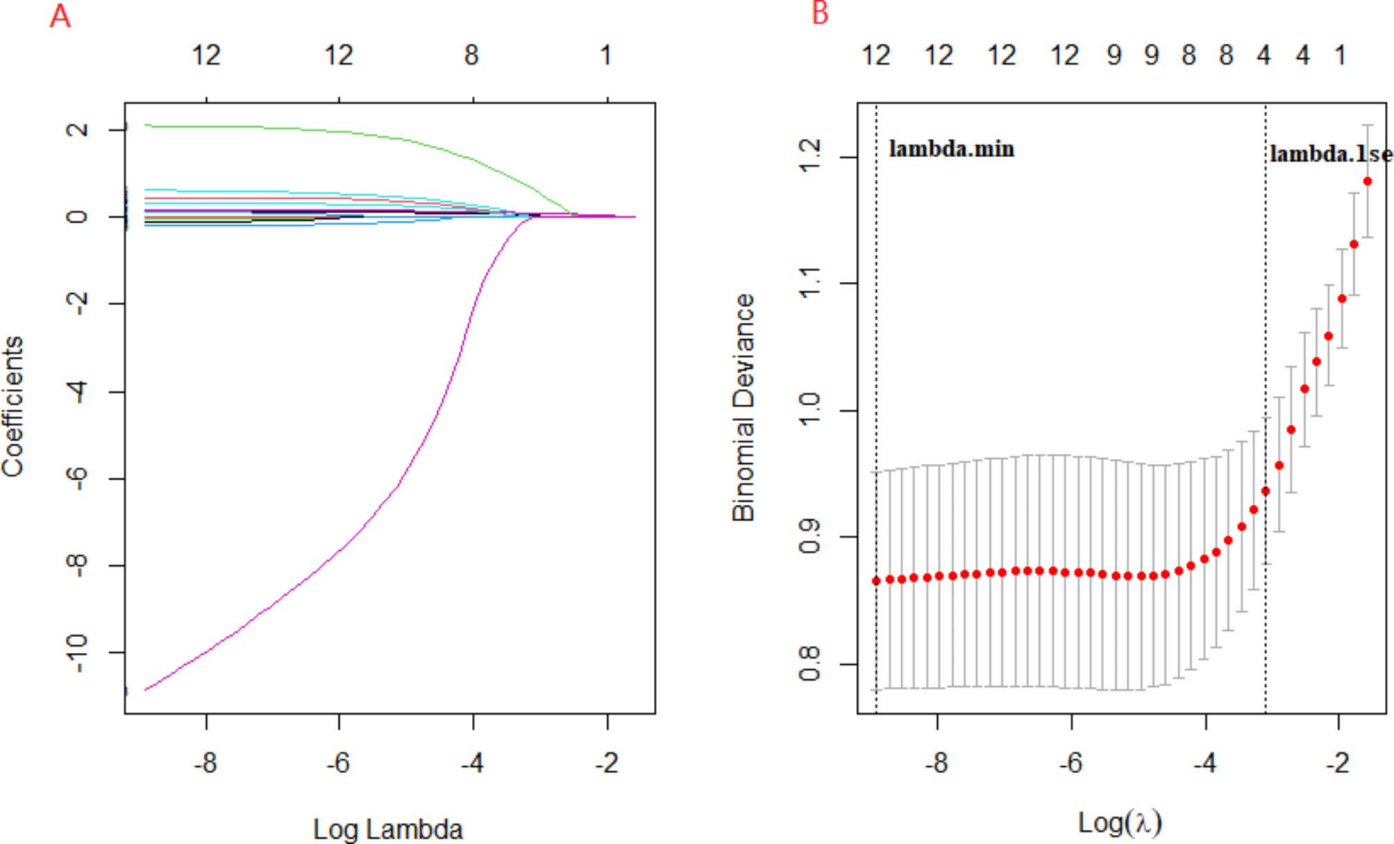
Predictors’ selection using LASSO regression method. **A** LASSO coefficient profiles of the 12 variables. The coefficient profile plot was produced against the log (λ) sequence. **B** The best penalty coefficient lambda was selected using a tenfold cross-validation and minimization criterion. By verifying the optimal parameter (λ) in the LASSO model, the binomial deviance curve was plotted versus log(λ) and dotted vertical lines were drawn based on 1 standard error criteria. 4 variables were selected based on 1 standard error

In univariate analysis, there were 12 variables with statistical significance between the two groups (**See** Table 1). The variables showing statistical significance by univariate analysis were included in the LASSO regression analysis (Fig. 1**)**, which showed that clinical course before bronchoscopy, length of stay, BMP formation, age were independent risk factors for long-term atelectasis in children.

### Development and validation of nomogram

**Fig. 2:**
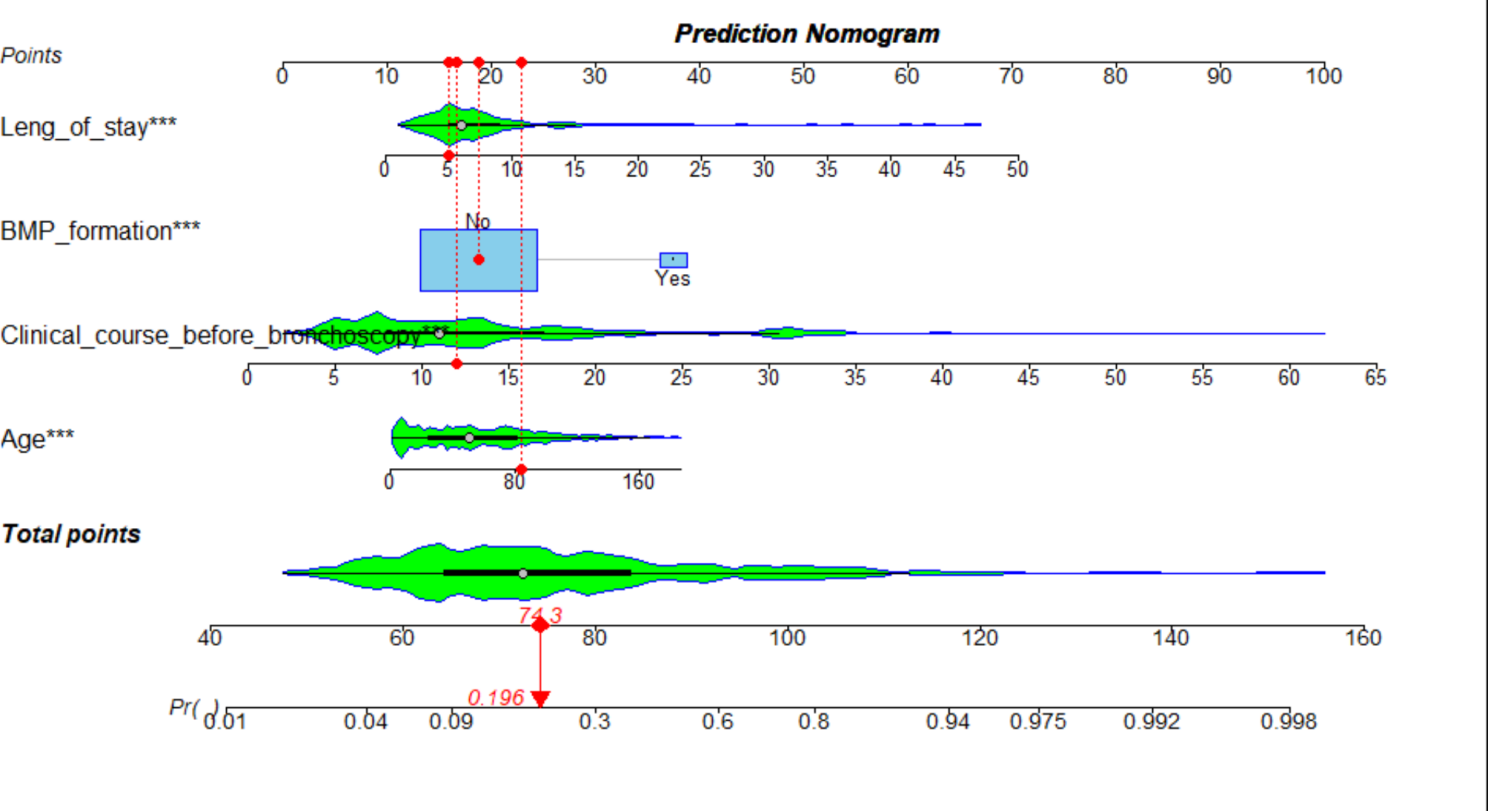
Nomogram was plotted based on four optimal predictors for long-term atelectasis in children. According to whether the cases had BMP formation, Clinical course before bronchoscopy ,Leng of stay and age, we can get a point on top lines. then add each point to obtain a total points and project it vertically on the bottom axis to obtain a predicted value of long-term atelectasis

**Fig. 3:**
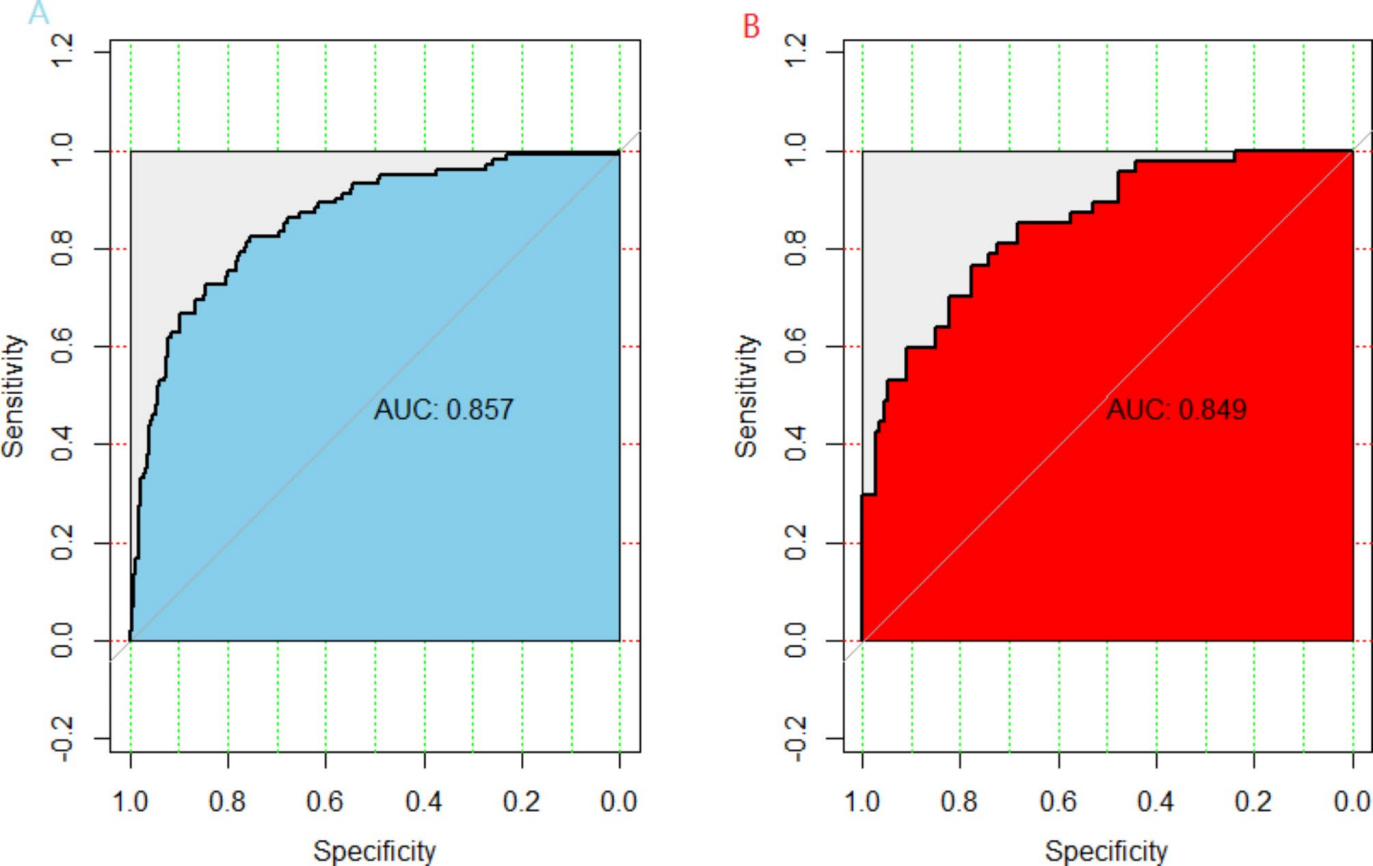
ROC curve of predictive nomogram. The sky-blue represents in the training set and the red represents the testing set

**Fig. 4:**
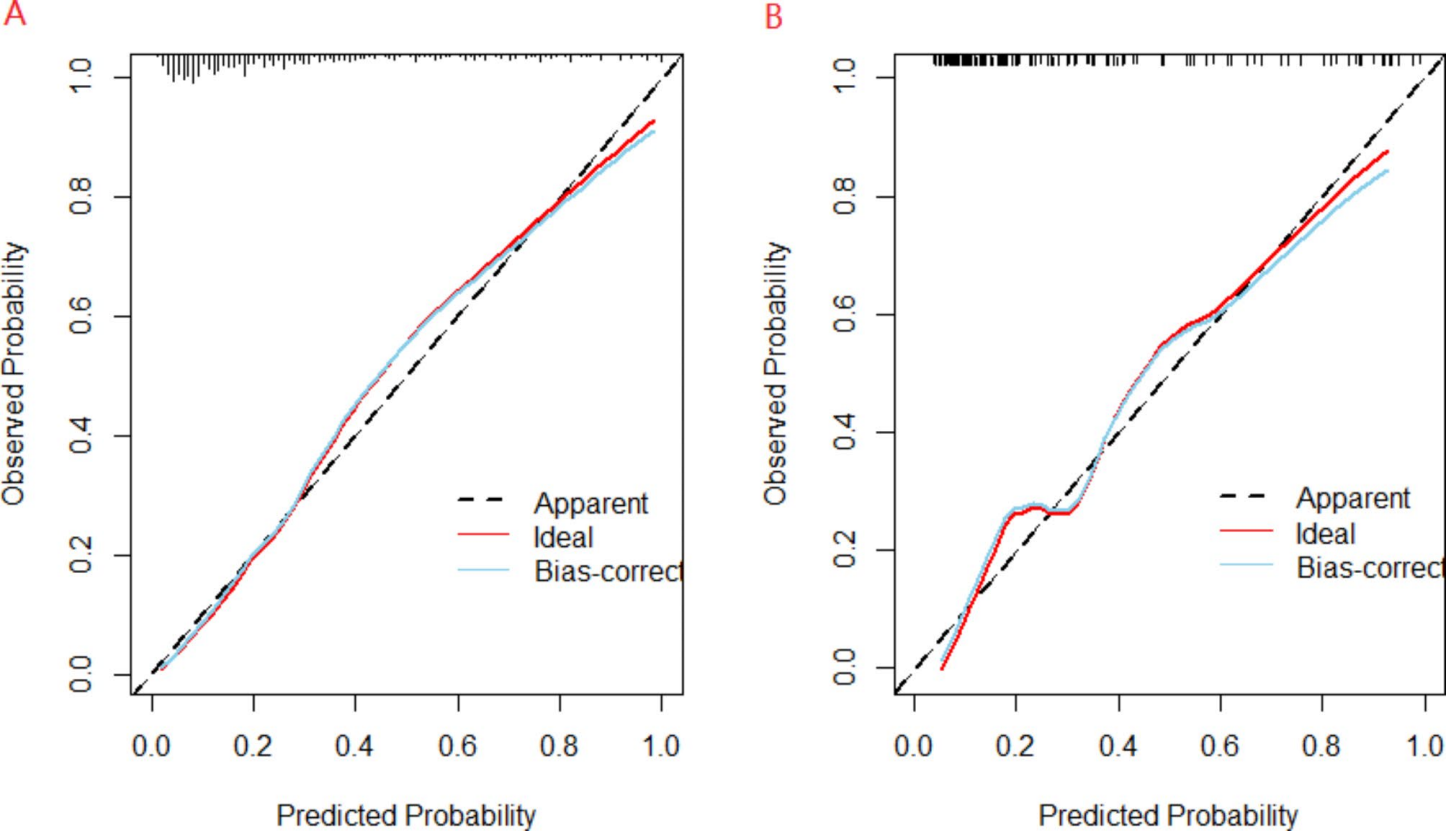
Calibration plots of the nomogram in (A) the training set and (B) the validating set

A prediction nomogram containing four independent predictors (clinical course before bronchoscopy, length of stay, BMP formation, age) was established by Logistic regression (Fig. 2). the logistic regression equation was logit(P)= (− 5.498) + 0.16X _clinical course before bronchoscopy_ + 0.142X _length of stay_ +0.167X _age_ + 1.699X _bronchial mucus plug formation_. For example, A patient with infective atelectasis who had BMP formation, had a Clinical course before bronchoscopy of 12 days, length of stay of 5 days, and age of 82 months, totaling 74.3(19 + 16.3 + 16 + 23) scores. The predicted risk is 0.196. In terms of diagnostic accuracy, the nomogram in the training and the test has good discrimination ability, with an AUC of 0.857(95% CI = 0.8136 ~ 0.9006) and 0.849(95% CI = 0.7848–0.9132) respectively (Fig. 3). In addition, 1000 Bootstrap resampling was used for internal validation. the calibration curves showed good consistency between observed probability and in probability in the training (Fig. 4-A) set and validating set (Fig. 4-B). Hosmer-Lemeshow test *P* values were 0.952(training set) and 0.763(validating set), respectively.

### Clinical utility of nomogram

**Fig. 5:**
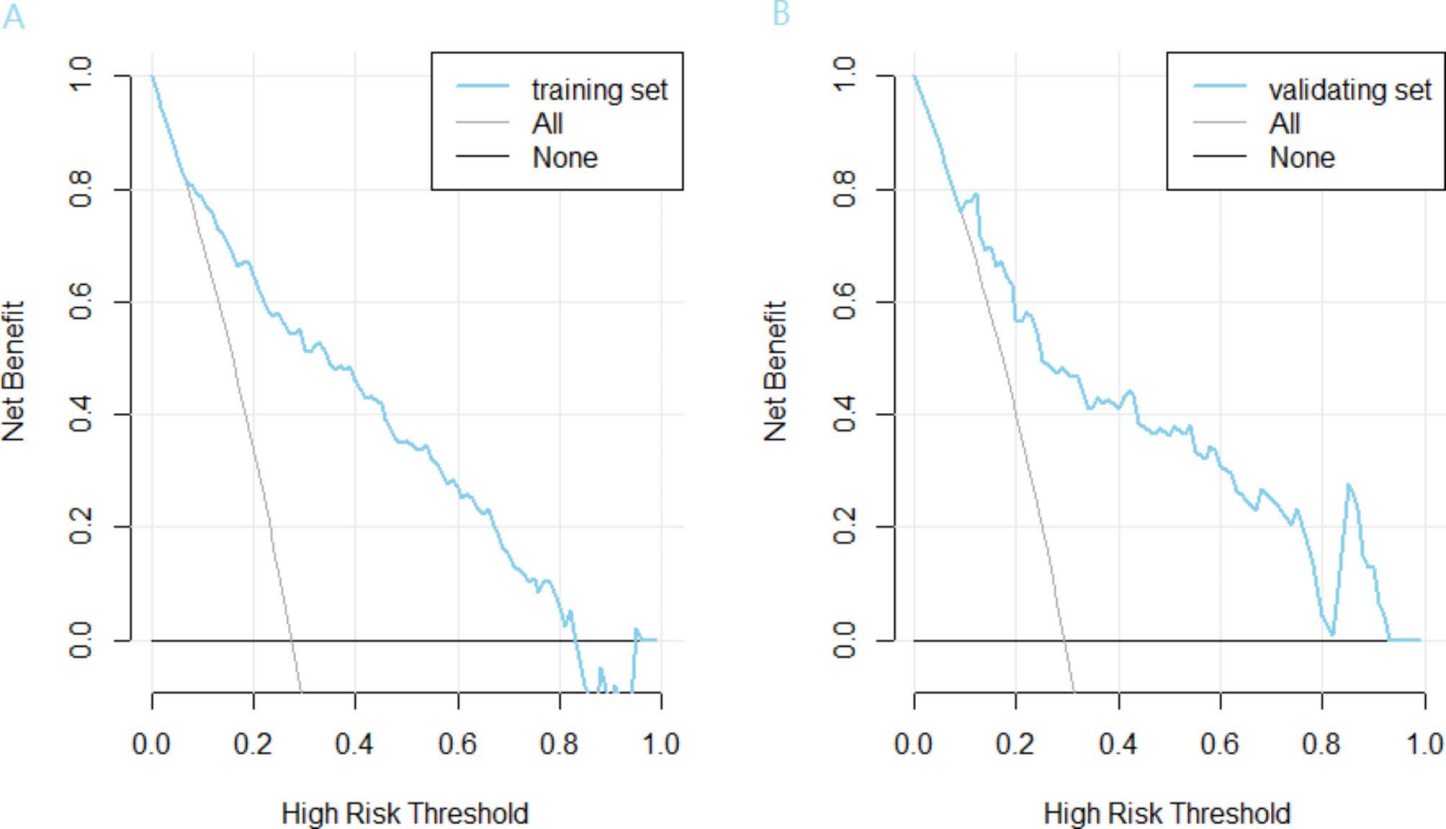
Decision curve analysis (DCA) for the predictive model. (A) the training set. (B) the validating set

The DCA curve is a novel method for evaluating the clinical utility of predictive models. Figure 5 showed that both the training set and the validation set had positive net benefit rates in the *Pt* range of about 0.2 ~ 0.8.

## Discussion

In this study, we retrospectively explored predictors of long-term atelectasis in children with pneumonia. Multivariate logistic analysis showed that clinical course before bronchoscopy, length of stay, BMP formation, age were optimal predictors of long-term atelectasis. The predictive nomogram constructed based on these four predictors showed good diagnostic accuracy, consistency and clinical utility.

In this study, BMP formation was risk factors for long-term atelectasis. It is well known that the formation of BMP is closely related to *Mycoplasma pneumoniae* infection [10]. BMP is considered to be a manifestation of severe abnormal cilia in the bronchus, which reduces the immune function of the airway and disrupts the clearance of mucocilia, causing mucus, inflammatory secretions, necrosis, bleeding, etc. to accumulate in the bronchus, forming endogenous bronchial foreign plugging bodies, leading to development of atelectasis [11]. Zhang et al. found that BMP could be used as an independent risk factor for predicting longer radiographic resolution in patients with refractory MMP [12]. The study of Zhao et al. [13]. also showed a close correlation between plastic bronchitis and atelectasis. Three studies attempted to identify risk factors or establish predictive models for the formation of BMP in patients with refractory RMMP [10, 11, 14]. Moreover, the infection rate of *Mycoplasma pneumoniae* in this study was statistically significant between the two groups. The fact that *Mycoplasma pneumoniae* infection is easy to BMP explained why Mycoplasma infection can easily lead to long-term atelectasis.

Bronchoscopy and BAL can control infection and promote pulmonary reexpansion by clearing the respiratory tract and improving drainage, and its clinical efficacy has been demonstrated in several studies [5, 7, 15]. And the timing of bronchoscopy is considered to have an important effect on the prognosis of atelectasis. Yan et al. found that interventional bronchoscopy after a clinical course of lasting ≥ 11.5 days before bronchoscopy was a risk factor for delayed imaging clearance in children, but the object of this study was *mycoplasma pneumoniae* infection. Our study also revealed that a longer clinical course before bronchoscopy was a risk factor of long-term infectious atelectasis, which may be associated with the fact that the longer the course of the disease, the more serious the destruction of the bronchial wall and irreversible structural remodeling are. In addition, a long course of disease often means delayed diagnosis and early empirical treatment because the pathogen is not clear. Drug use based on the results of drug sensitivity test is the most ideal method for the treatment of pneumonia, but if there are no clear etiological results, clinicians often use drugs according to experience, which can easily lead to the abuse of antibiotics and the increase of drug-resistant bacteria. It is well known that the extraction of samples from the lower respiratory tract through bronchoscopy is the most ideal way to find the pathogen of pneumonia. These suggest that once atelectasis is diagnosed, bronchoscopy and BAL may be performed as early as possible to reduce the risk of multiple bronchoscopy and improve the therapeutic effect of BAL.

This study also found that long-term atelectasis was related to age. We conjectured that it might be due to the different positions of atelectasis in patients of different age groups. Previous studies [16] have found that atelectasis in patients over 3 years old is more concentrated in the middle and lower lobe, while infants have a higher proportion of upper lobe atelectasis, which is consistent with our research conclusions. This can be explained by the fact that children are more likely to maintain an upright position whereas infants are usually in a supine position. Atelectasis of upper lobe is not conducive to clearing the respiratory tract and improving drainage through BAL, as well as difficulties in infant care may be the causes of long-term atelectasis. In addition, it is reasonable that the longer the hospital stay, the more serious the condition, the greater the possibility of long-term atelectasis.

This study has some limitations. First, this was a single center, retrospective study, which can easily lead to selection deviation. Secondly, this study did not have a long-term follow-up on the prognosis of children with atelectasis. Finally, external validation data for model validation was lacking in this study.

## Conclusion

This nomogram model based on the risk factors of long-term atelectasis in children with pneumonia has good accuracy and consistency, and can provide some reference value for clinical prevention and treatment of long-term atelectasis in children.

## Data Availability

The datasets generated and/or analyzed during the current study are not publicly available due our research center policy, but are available from the corresponding author on reason-able request.

## References

[1] Peroni DG, Boner AL (2000). Atelectasis: mechanisms, diagnosis and management. Paediatr Respir Rev.

[2] Ullmann N, D’Andrea ML, Gioachin A (2020). Lung ultrasound: a useful additional tool in clinician’s hands to identify pulmonary atelectasis in children with neuromuscular disease. Pediatr Pulmonol.

[3] Faro A, Wood RE, Schechter MS (2015). Official american thoracic Society technical standards: flexible airway endoscopy in children. Am J Respir Crit Care Med.

[4] Wang L, Xie Q, Xu S et al. The role of flexible bronchoscopy in children with Mycoplasma pneumoniae pneumonia. Pediatr Res 2022. 10.1038/s41390-021-01874-z 35459766

[5] Yang M, Yang DH, Yang X, Wang YS, Wu L, Chen ZM (2018). [Efficacy of bronchoalveolar lavage and its influence factors in the treatment of Mycoplasma pneumoniae pneumonia with atelectasis]. Zhonghua Er Ke Za Zhi.

[6] Li F, Zhu B, Xie G, Wang Y, Geng J. Effects of bronchoalveolar lavage on pediatric refractory mycoplasma pneumoniae pneumonia complicated with atelectasis: a prospective case-control study. Minerva Pediatr. 2020. 10.23736/S2724-5276.20.05538-3 32241100

[7] Zhang Y, Chen Y, Chen Z (2014). Effects of bronchoalveolar lavage on refractory Mycoplasma pneumoniae pneumonia. Respir Care.

[8] Su D-Q, Li J-F, Zhuo Z-Q (2020). Clinical analysis of 122 cases with Mycoplasma Pneumonia complicated with atelectasis: a retrospective study. Adv Ther.

[9] McIntosh K (2002). Community-acquired pneumonia in children. N Engl J Med.

[10] Zhang J, Wang T, Li R (2021). Prediction of risk factors of bronchial mucus plugs in children with Mycoplasma pneumoniae pneumonia. BMC Infect Dis.

[11] Xu Q, Zhang L, Hao C (2017). Prediction of bronchial mucus plugs formation in patients with Refractory Mycoplasma Pneumoniae Pneumonia. J Trop Pediatr.

[12] Huang L, Huang X, Jiang W, Zhang R, Yan Y, Huang L (2018). Independent predictors for longer radiographic resolution in patients with refractory Mycoplasma pneumoniae pneumonia: a prospective cohort study. BMJ Open.

[13] Zhao L, Zhang T, Cui X (2022). Development and validation of a nomogram to predict plastic bronchitis in children with refractory Mycoplasma pneumoniae pneumonia. BMC Pulm Med.

[14] Xu X, Li H, Sheng Y (2020). Nomogram for Prediction of bronchial mucus plugs in children with Mycoplasma pneumoniae Pneumonia. Sci Rep.

[15] Li F, Zhu B, Xie G, Wang Y, Geng J (2021). Effects of bronchoalveolar lavage on pediatric refractory mycoplasma pneumoniae pneumonia complicated with atelectasis: a prospective case-control study. Minerva Pediatr (Torino).

[16] Marini JJ (2019). Acute Lobar Atelectasis. Chest.

